# The Changes of Cholesterol Profile at the Different Insulin Resistance Range in the Czech Republic

**DOI:** 10.3390/medicina57030249

**Published:** 2021-03-08

**Authors:** Vladimír Kron, Miroslav Verner, Pavel Smetana, Dagmar Horáková, Jan Šlégr, Filip Studnička, Damián Bušovský, Karel Martiník

**Affiliations:** 1 Department of Food Biotechnologies and Agricultural Products Quality, Faculty of Agriculture, University of South Bohemia, Studentská 1668, 370 05 České Budějovice, Czech Republic; vladimir.daniel.kron@gmail.com (V.K.); pavel.smetana@uhk.cz (P.S.); 2 Ambulance for Metabolic Assessment of Prof. MUDr. Karel Martiník, DrSc., s.r.o., Bratří Štefanů 895, 500 03 Hradec Králové, Czech Republic; karel.martinik@seznam.cz; 3 Department of Clinical Biochemistry, Hospital of České Budějovice, B. Němcové 585/54, 370 01 České Budějovice, Czech Republic; verner@nemcb.cz; 4 Faculty of Health and Social Sciences, Institute of Laboratory Diagnostics, University of South Bohemia, J. Boreckého 1167/27, 370 11 České Budějovice, Czech Republic; 5 Department of Public Health, Faculty of Medicine and Dentistry, Palacký University Olomouc, Hněvotínská 3, 775 15 Olomouc, Czech Republic; dagmar.horakova@upol.cz; 6 Center of Advanced Technology, Faculty of Science, University of Hradec Králové, Roktianského 62, 500 03 Hradec Králové, Czech Republic; filip.studncika@uhk.cz (F.S.); damian.busovsky@uhk.cz (D.B.)

**Keywords:** lipoprotein, HDL, LDL, glucose, insulin, HOMA IR

## Abstract

*Background and Objectives:* The mechanism of the relationship between glycemia and lipid metabolism has not been completely clarified, and slight differences may be found between authors and the kinds of evaluated parameters. Therefore, this study focused on possible changes of lipoprotein profile with regards to HOMA IR (Homeostatic Model Assessment for Insulin Resistance) cut-off 3.63, considered a signal of glucose metabolism alterations. *Materials and Methods:* The metabolic profiles of 3051 individuals were divided by HOMA IR values into two groups below cut-off 3.63, including (*n* = 2627) and above cut-off (*n* = 424). Patients taking medication or supplements to affect lipid, insulin, or glucose metabolism were excluded. Fasting glucose levels, insulin, and lipoproteins (total, HDL—high density and LDL—low density lipoprotein cholesterol) were compared between the groups with different HOMA IR. After analysis of data distribution, *F*-test and *t*-test were provided to compare variances and mean values. *Results:* The evaluation shows that the kind of cholesterol is crucial for a possible relationship with glucose metabolism and consequently confirms the changes of lipoproteins (HDL and LDL) by HOMA IR cut-off 3.63. *Conclusions:* The results of patients divided by HOMA IR cut-off 3.63 also suggest possible changes in the regulation of glucose metabolism and lipoprotein concentrations (HDL and LDL).

## 1. Introduction

The relationship between lipid metabolism and insulin resistance, has been the objective of medical trials, mostly in the last decade. Although it is possible to find an incidence of changing lipid parameters and insulin resistance in patients with metabolic syndrome, this association hasn’t been clarified and seems to be more complicated [1]. Some trials have described the influence of cholesterol, triglycerides, or free fatty acids on insulin secretion, resulting in the change of glycemia parameters. These conclusions have not appeared only in patients with metabolic syndrome and with normal toleration of glucose (NGT individuals). On the other hand, some studies dealing with glycemia principles claim that glucose and lipid metabolism parameters should be considered independently.

Zheng et al. evaluated the associations between dyslipidemia, insulin resistance, and function of β cells in normal glucose-tolerated patients and the population with glucose impaired regulation. The trial was established to prove if dyslipidemia could affect β cell function, especially in nondiabetic subjects [2]. Bardini et al. submitted a similar hypothesis and confirmed that cholesterol homeostasis should be evaluated as an essential factor of adequate insulin secretion and optimal β cells performance [3]. Ikeoka et al. and Díaz-Ruiz et al. also suggested that the monitoring of triglycerides and cholesterols is a useful tool to determine the concomitant occurrence of insulin resistance. Furthermore, these authors recommend analyzing this relationship in additional population studies with practical results for lipid metabolism and insulin resistance indication [4,5]. Nagel et al. concluded that lipid metabolism should be a part of the metabolic profile, especially at the period of insulin resistance therapy [6]. Furuhashi et al. also focused on an evaluation of free fatty acids and cholesterol concentrations and claimed that high levels of both are a common feature of insulin-resistant states [7].

## 2. Materials and Methods

### 2.1. Parameters for Evaluation

The evaluation of the HOMA IR score (Homeostasis Model Assessment for Insulin Resistance) has been recommended not only for the patients with insulin resistance but also without the symptoms of adverse glucose metabolism [8]. Insulin resistance was measured by HOMA IR score based on insulin and glucose concentration according to the methodology of the glucose tolerated test (OGTT) [8,9]. HOMA IR with cut-off 3.63 has been established for both genders to divide the groups with relevant lipid and glycaemia parameters [10]. HDL (high density lipoprotein), LDL (low density lipoprotein), and total cholesterol concentrations contributed to the investigation of lipoprotein metabolism [11]. Díaz-Ruiz et al. used a similar methodology to describe hydro-carbonated alterations at different fractions of cholesterols [5].

### 2.2. Patients and Samples

Metabolic profiles were collected in the Czech Republic from 2009 until 2017, and all participants (*n* = 3051) gave their consent to anonymous data analysis. The patients included were aged between 15 and 78 years to ensure an optimal profile of the population. The individuals receiving therapy of lipid or glucose metabolism were excluded, and this limitation also included the supplements to affect insulin, glucose, or lipid concentration. All samples were processed correctly (centrifugation, serum preparation, etc.) and analyzed.

All parameters (age, glucose, insulin, HOMA IR, cholesterols) were divided into two subgroups: subjects with HOMA IR Index ≤3.63 (*n* = 2627) and >3.63 (*n* = 424) had to be relevant for individual patients. HOMA IR cut-off was set at 3.63 and considered a signal of glucose metabolism alterations within the population [10].

### 2.3. Laboratory Analysis

All serum and plasma biochemical parameters were determined from venous blood. Insulin concentrations were analyzed by regulatory impact assessment methodology from the plasma with the use of immunochemical methods. Glucose concentrations were measured enzymatically as well as total cholesterol (enzymatic assays). Homogeneous methods were used for the detections of HDL and LDL in an auto-analyzers. All biochemical parameters for tests were provided by the instructions of manufacturers (Roche Diagnostics, Basel and Abbott Laboratories, Abbott Park, Illinois) with a strict following of the measurement continuity [12,13].

### 2.4. Statistical Analysis

At first, basic statistical parameters were calculated for all groups: number of values, arithmetic mean ($\bar{x}$), standard deviation ($s_{x}$), variance (σ), minimum (Min) and maximum (Max.). Lower whiskers, upper whiskers, quartiles, and medians identified data distribution. The *F*-test (with quantile *F*_0.975_) was performed to test the equality of variance and select the right type of *t*-test. A minimal level of statistical significance (*H_0_*:*H_a_*) was set at *p* < 0.05. The normality and the possible convergence were not limiting because the right types of *t*-test processes mean values reached in tens as a normal distribution (central limit theorem) [13]. All statistical parameters were calculated using Microsoft Excel (raw data) (Microsoft, Redmond, WA, USA) and Statistica (AnalystSoft, Alexandria, VA, USA) and StatPlus (AnalystSoft, Alexandria, VA, USA) software. The data file was not divided by gender, and critical values of HOMA IR were set for both genders and not separately [10].

## 3. Results

**Figure 1:**
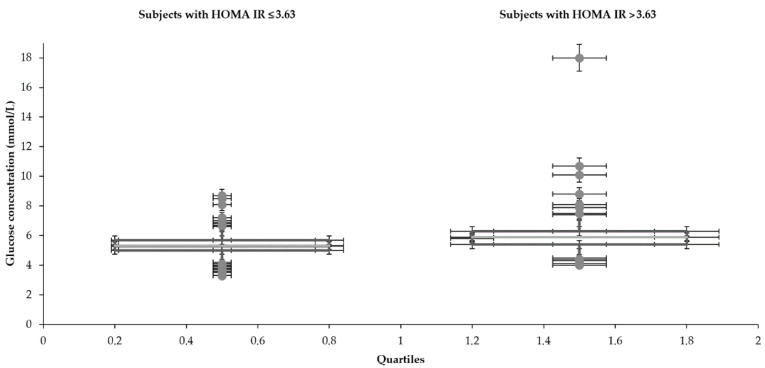
Distribution of glucose with the different range of HOMA IR.

**Figure 2:**
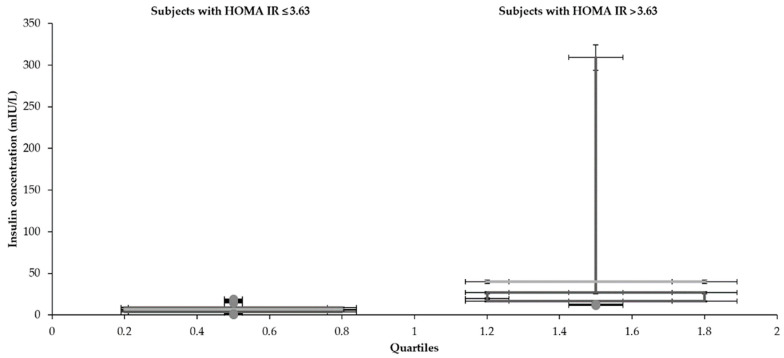
Distribution of insulin with the different range of HOMA IR.

**Figure 3:**
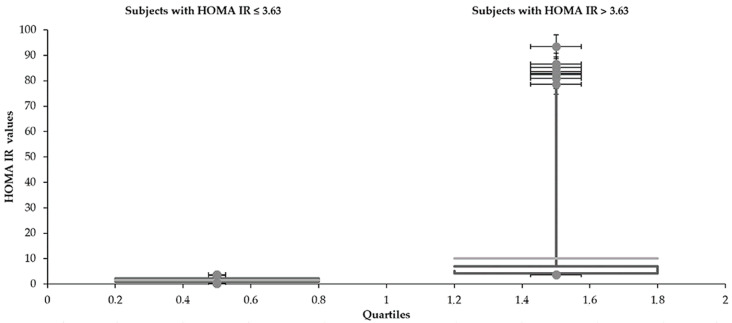
Distribution of insulin with the different range of HOMA IR.

**Figure 4:**
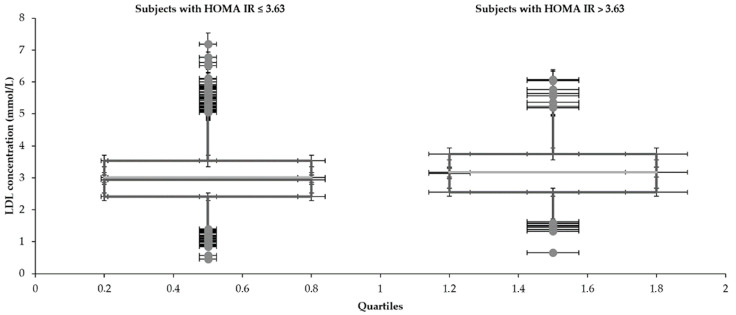
Distribution of LDL with the different range of HOMA IR.

**Figure 5:**
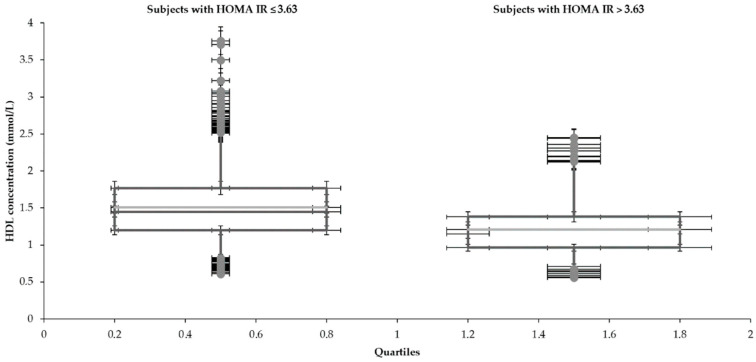
Distribution of HDL with the different range of HOMA IR.

**Figure 6:**
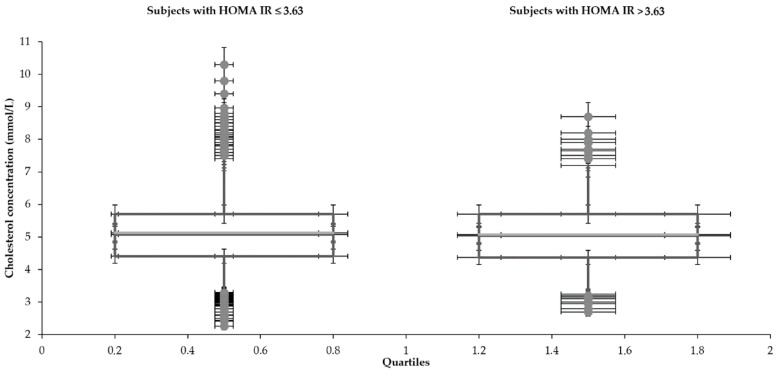
Data distribution of total cholesterol with the different range of HOMA IR.

Statistical characteristics of groups divided by HOMA IR 3.63 are shown in Table 1 and Table 2 and Figure 1, Figure 2, Figure 3, Figure 4, Figure 5 and Figure 6. The mean values for the groups with various HOMA IR range with the optimal ranges are described in Table 3. Data distribution of parameters divided by HOMA IR 3.63 is included in Table 4.

The mean glucose concentrations (5.3 and 5.9 mmol/L) fluctuated around the upper limit of the physiological range (5.6 mmol/L) regardless of the HOMA IR cut-off. Cholesterols also reached slightly higher values (total 5.1 mmol/L—both groups), although the oscillation was more balanced. In comparison to the group with an average HOMA IR 1.6, increased concentrations of glucose and insulin were naturally found in the subjects with a higher mean value of HOMA IR (10.1). Significantly, insulin (6.7 and 39.8 mIU/L) and glucose (5.3 and 5.9 mmol/L) differed in individuals divided by the HOMA IR 3.63. A broader scale of data was recorded in glycemia metabolites too. Both glucose (3.3–8.7 and 4.0–18.0 mmol/L) and insulin level (0.6–19.4 and 11.5–309.0 mIU/L) reached a more comprehensive range of values in the patients with increased HOMA IR (above 3.63). The LDL concentration was higher in the group with HOMA IR above 3.63 (3.0 and 3.2 mmol/L). The HDL cholesterol showed the opposite tendency (1.5 and 1.2 mmol/L). The individuals were aged between 15–73 despite different intervals of the HOMA IR 3.63.

## 4. Statistical Analysis

The *F*-test determined the equality of variances between the groups divided by HOMA IR cut-off 3.63. Statistically significant differences (*p* = 0.05) of cholesterol variances (total, HDL, and LDL, Table 5) were calculated between the groups with the HOMA IR (above and below 3.63, including).

As follows, the *t*-test was used for unequal variances to compare mean values between groups with the different HOMA IR range (cut-off 3.63). HDL and LDL cholesterol reached statistically significant differences between groups with the HOMA IR 3.63 (Table 6).

## 5. Discussion

The results of glycemia and insulin resistance in the group below and including HOMA IR 3.63 corresponded with the metabolic profiles of nondiabetic patients provided by Horáková et al. in the Czech Republic (glucose 5.3 and 5.4 mmol/L, insulin 6.7 and 9.1 mIU/L, HOMA IR 1.6 and 1.7). Equally, lipoprotein parameters reached similar values in the same study (cholesterols—total 5.1 and 5.1, HDL 1.5 and 1.5, LDL 3.0 and 3.0 mmol/L) [10]. Variances of cholesterols (total, HDL, LDL) were statistically significant between the groups with different HOMA IR (above and below 3.63 included). The mean values test showed that especially LDL cholesterol is an important parameter, which changes by HOMA IR cut-off 3.63 (3.0 and 3.2 mmol/L). The opposite tendency appeared in HDL concentrations (1.5 and 1.2 mmol/L), which increased in the group with a lower range of HOMA IR (below and including 3.63). Generally, this trial’s results suggested that HOMA IR values could signal lipid parameter changes.

Díaz-Ruiz et al. came to a similar conclusion in a Valencia region trial in which glucose concentrations positively correlated with total glyceride and LDL level. Equally, HDL concentration showed to be negatively correlated with insulin level, which could subsequently affect HOMA IR values [5]. Zheng et al. also confirmed that HOMA IR values could positively affect LDL and total glyceride concentrations and negatively affect HDL in regular glucose-tolerating patients [2]. Mora et al. found the association of lipoproteins in participants with the risk of diabetes stratified by a higher level of LDL (median 121 mg/dL or 3.1 mmol/L) [15]. Day also warned that higher insulin resistance (e.g., HOMA IR) increased the risk of hyperlipidemia [16]. With exception to individual parameters for glycemia and lipid metabolism, Toro-Huamanchumo et al. recommended evaluating this relationship according to the TGI Index (ratio triglycerides/glucose). Elevated TGI (≥8.65) was associated with insulin resistance and hyperinsulinemia during an oral glucose tolerance test in healthy adults [17]. Keska et al. assessed the variability of HOMA IR and lipoprotein profile in Poland’s young active men. The results of subjects with HOMA IR above 1.34 (median) showed a significant and positive correlation with total and LDL cholesterol [18]. Lee et al. recorded a similar trend of glycemia and cholesterol values but with lower HOMA IR cut-off (1.4 and 2.0) in the Chinese population with normal glucose tolerant metabolism. There was a higher level of total (5.0 and 5.4 mmol/L) and LDL cholesterols (1.2 and 1.3 mmol/L) in patients with the range of HOMA IR 1.1–2.7. However, HDL concentration was decreased (1.2 and 1.1 mmol/L). A similar tendency appeared in the quartiles of subjects with HOMA IR above 1.84 (Q4) when total and LDL cholesterols were also increased (5.1 and 5.3, 3.3, and 3.4 mmol/L). HDL concentration was slightly lower (1.2 and 1.1 mmol/L) in the patients with higher HOMA IR range 1.1–1.8 [19].

Reversely, Parhofer reported a relationship of lipid and glycemic values and consequently mentioned that lipoprotein and glucose concentrations should be considered separately, not directly to each other. This statement was established on lipoprotein levels, which were weakly correlated with other lipid abnormalities [1]. Shalaurova et al. submitted similar conclusions. Measured lipoproteins and insulin resistance index values (LP-IR) exhibited stronger associations with HOMA IR than each of the individual parameters [20]. Notsu et al. also recorded the correlation between HOMA IR and specific types of cholesterols (HDL) and referred to the importance of dividing lipoprotein parameters for possible determinations [21].

## 6. Limitations of the Study

There was an unbalanced number of participants in the groups divided by HOMA IR cut-off 3.63. Higher numbers of individuals with HOMA IR above 3.63 could be optimal to reach better data distribution. HDL concentrations are located more around the mean value. Therefore, the variability of this group is lower. The study doesn’t include complete changes in the lipoprotein spectrum or the influence of physical activity.

## 7. Conclusions

The glycemia evaluation has recently expanded to other metabolic assessments (lipid, thyroid, or lymphatic system functions). This study’s results consider the importance of what type of lipoprotein parameter should be used for evaluation and how it will affect the possible relationship. HOMA IR cut-off 3.63 was determined not only as a useful indicator of establishing insulin resistance but also as a potential signal of lipoprotein alterations. HOMA IR cut-off 3.63 seemed to be relevant regarding HDL and LDL concentrations, as well as the variances of all lipoproteins. Overall, the HOMA IR appears to be a useful tool for early preventive procedures within lipid or glucose metabolism, especially concerning the increase of cardiovascular and metabolic diseases.

## Figures and Tables

**Table 1:** Statistical characteristics of the individuals with HOMA IR ≤ 3.63.

Characteristics	Age (y)	Glucose (mmol/L)	Insulin (mIU/L)	Total Cholesterol (mmol/L)	HDL Cholesterol (mmol/L)	LDL Cholesterol (mmol/L)	HOMA IR
*N*	2627	2627	2627	2627	2627	2627	2627
$\bar{x}$	40.3	5.3	6.7	5.1	1.5	3.0	1.6
$s_{x}$	11.5	0.6	3.5	1.0	0.4	0.9	0.8
Σ	132.9	0.3	11.9	1.0	0.2	0.8	0.7
Min	15.0	3.3	0.6	2.3	0.6	0.5	0.1
Max	78.0	8.7	19.4	10.3	3.8	7.2	3.63

**Table 2:** Statistical characteristics of the individuals with HOMA IR >3.63.

Characteristics	Age (y)	Glucose (mmol/L)	Insulin (mIU/L)	Total Cholesterol (mmol/L)	HDL Cholesterol (mmol/L)	LDL Cholesterol (mmol/L)	HOMA IR
*N*	424	424	424	424	424	424	424
$\bar{x}$	41.5	5.9	39.8	5.1	1.2	3.2	10.1
$s_{x}$	12.6	1.0	68.9	1.0	0.3	0.9	16.9
Σ	159.4	0.9	4749.2	1.0	0.1	0.8	284.2
Min	15.0	4.0	11.5	2.7	0.6	0.75	3.6
Max	73.0	18.0	309.0	8.7	2.5	6.1	93.4

**Table 3:** Comparison of mean values between the groups with various HOMA IR range with the optimum.

Characteristics	Age (y)	Glucose (mmol/L)	Insulin (mIU/L)	Cholesterols (mmol/L)			HOMA IR
				Total	HDL	LDL	
Subjects with HOMA IR ≤ 3.63	40.3	5.3	6.7	5.1	1.5	3.0	1.6
Subjects with HOMA IR > 3.63	41.5	5.9	39.8	5.1	1.2	3.2	10.1
Optimal range of values [9,14]	-	3.9–5.6	2.5–24.0	2.9–5.0	1.0–2.1	1.2–3	<3.63

**Table 4:** Data distribution of testing parameters divided by HOMA IR 3.63.

**Glucose (mmol/L)**					
Subjects with HOMA IR	Lower whisker	Q1	Median	Q3	Upper whisker
≤3.63	4.2	5.0	5.3	5.7	6.6
>3.63	4.5	5.4	5.8	6.3	7.4
**Insulin (mIU/L)**					
Subjects with HOMA IR	Lower whisker	Q1	Median	Q3	Upper whisker
≤3.63	2.00	4.0	6.0	9.0	14.8
>3.63	13.2	16.5	19.7	26.8	309.0
**HOMA IR**					
Subjects with HOMA IR	Lower whisker	Q1	Median	Q3	Upper whisker
≤3.63	0.4	0.9	1.4	2.1	3.5
>3.63	3.7	4.2	5.1	7.0	78.3
**LDL (mmol/L)**					
Subjects with HOMA IR	Lower whisker	Q1	Median	Q3	Upper whisker
≤3.63	1.4	2.4	2.9	3.5	5.0
>3.63	1.6	2.6	3.1	3.8	5.2
**HDL (mmol/L)**					
Subjects with HOMA IR	Lower whisker	Q1	Median	Q3	Upper whisker
≤3.63	0.8	1.2	1.5	1.8	2.5
>3.63	0.7	1.0	1.2	1.4	2.1
**Total cholesterol (mmol/L)**					
Subjects with HOMA IR	Lower whisker	Q1	Median	Q3	Upper whisker
≤3.63	3.3	4.4	5.1	5.7	10.3
>3.63	3.2	4.4	5.0	5.7	8.7

HDL, high density lipoprotein; LDL, low density lipoprotein; HOMA IR Index, Homeostasis Model Assessment of Insulin Resistance.

**Table 5:** Results of *F*-test for the different ranges of HOMA IR (≤3.63 and >3.63).

Parameter	Total Cholesterol (mmol/L)	HDL Cholesterol (mmol/L)	LDL Cholesterol (mmol/L)
***F* critical value _(*p* = 0.05)_**	0.89	0.89	0.89
***p*-value**	0.005	0.006	0.004
***F*-test**	0.956 *	1.621 *	0.946 *

* 0.05 < *p*; HDL: high density lipoprotein; LDL: low density lipoprotein.

**Table 6:** Results of *t*-test for the different ranges of HOMA IR (≤3.63 and >3.63).

Parameter	Total Cholesterol (mmol/L)	HDL Cholesterol (mmol/L)	LDL Cholesterol (mmol/L)
***T* critical value _(*p* = 0.05)_**	1.96	1.96	1.96
***p*-value**	0.96	0.00001	0.00002
***T* test**	0.51	13.86 **	4.77 **

** *p* < 0.0001; HDL: high density lipoprotein; LDL: low density lipoprotein.

## Data Availability

The datasets generated for this study are available on request to the corresponding author.
